# *In vivo* mechanical characterization of arterial wall using an inverse analysis procedure: application on an animal model of intracranial aneurysm

**DOI:** 10.1098/rsos.231936

**Published:** 2024-04-17

**Authors:** J. Raviol, G. Plet, J. B. Langlois, S. Si-Mohamed, H. Magoariec, C. Pailler-Mattei

**Affiliations:** ^1^ Ecole Centrale de Lyon, CNRS, ENTPE, LTDS, UMR 5513, Écully 69130, France; ^2^ CERMEP - Imagerie du Vivant, Bron 69500, France; ^3^ Université de Lyon, INSA Lyon, Université Claude Bernard Lyon 1, UJM-Saint Etienne, CNRS, Inserm, CREATIS UMR 5220, U1206, F69621, Villeurbanne 69100, France; ^4^ Département de Radiologie, Hôpital Louis Pradel, Hospices Civils de Lyon, Bron 69677, France; ^5^ Université de Lyon, Université Claude Bernard Lyon 1, ISPB-Faculté de Pharmacie, Lyon 69008, France

**Keywords:** finite element method, patient-specific numerical modelling, fluid–structure interaction, luminal volume variation, spectral photon computed counting tomography

## Abstract

Intracranial aneurysm is a pathology related to the deterioration of the arterial wall. This work is an essential part of a large-scale project aimed at providing clinicians with a non-invasive patient-specific decision support tool to facilitate the rupture risk assessment. It will lean on the link between the aneurysm shape clinically observed and a database derived from the *in vivo* mechanical characterization of aneurysms. To supply this database, a deformation device prototype of the arterial wall was developed. Its use coupled with medical imaging (spectral photon-counting computed tomography providing a spatial resolution down to 250 μm) is used to determine the *in vivo* mechanical properties of the wall based on the inverse analysis of the quantification of the wall deformation observed experimentally. This study presents the *in vivo* application of this original procedure to an animal model of aneurysm. The mechanical properties of the aneurysm wall identified were consistent with the literature, and the errors between the numerical and experimental results were less than 10%. Based on these parameters, this study allows the assessment of the aneurysm stress state for a known solicitation and points towards the definition of a rupture criterion.

## 1. Introduction

An intracranial aneurysm (IA) constitutes an irreversible and structural deformation of the cerebral artery wall. It is considered a public health concern. In the global population, the estimated prevalence of IA is 2–5% [1], and the annual risk of rupture for those affected ranges from 1% to 4% [2–4]. Subarachnoid haemorrhage (SAH) resulting from the rupture of IA is associated with a mortality rate of 30–40% [5]. Survivors may encounter functional dependence (one in five cases) as well as psychological and neurological complications if they regain independence [6–9]. Unruptured intracranial aneurysms (UIAs) are usually detected fortuitously through routine examinations. The optimal approach to managing these aneurysms can depend on either the aneurysm’s morphological characteristics (such as its location, size and shape) or the patient’s epidemiological factors (such as age and medical history) [10–13]. Associated scores such as PHASES (Population, Hypertension, Age, Size of the aneurysm, Earlier SAH from other aneurysms, Site of aneurysm) and UIATS (Unruptured Intracranial Aneurysm Treatment Score) are mainly used in treatment decisions [4,14,15]. In addition, it is important to consider the risks associated with medical procedures such as endovascular treatments or surgical clipping [8]. While there is a general consensus that large UIAs should be considered for surgical treatment [16], there remains no agreement on the optimal management of other types of aneurysms. Furthermore, the mechanical condition of the aneurysm wall is not factored into the aforementioned scoring systems, although it is believed to play an essential role in assessing rupture risk [17]. There is currently no method for predicting the rupture risk based on the *in vivo* quantitative determination of the aneurysm wall stress state. The experimental investigations of UIA mechanical characterization have been done only on *ex vivo* tissue samples using uniaxial tensile tests [18,19], biaxial tensile tests [20] and indentation tests [21]. The numerical modelling of the IA haemodynamics enables studying patient-specific cases, focusing on the fluid–structure interaction (FSI) between the blood flow and the artery wall [22–24] and considering several anatomical sizes and variations of the IA and brain circulatory system [25,26].

The present work is a key element of a large-scale project dedicated to the *in vivo* characterization of the mechanical properties of UIA. The long-term objective is to provide clinicians with a non-invasive decision tool to predict the rupture of an IA from a standard clinical image. The probability of rupture will be determined from a machine learning algorithm linking the UIA shape clinically observed and a database containing UIA clinical images associated with *in vivo* mechanical properties and rupture characterization. These specific properties, constituting the basis of this procedure, were derived from an original and non-destructive mechanical characterization device based on the aneurysm’s *in vivo* deformation. This deformation device prototype (DDP) was developed and calibrated on polymeric phantom arteries, depicting several testing conditions prior to the *in vivo* application on small animals [27]. On the strength of these conclusions and the *in vitro* proof of concept, this study depicted the *in vivo* application of the device on small animals and the original numerical procedure leading to the *in vivo* mechanical characterization of the aneurysm. The keystone of this procedure is the measurement of the aneurysm deformation owing to the effect of the DDP. This is done using the prototype spectral photon-counting computed tomography (SPCCT; Philips Healthcare) system: this new and promising clinical imaging technique provides a spatial resolution down to 250 μm [28–30], which was essential to visualize the deformation of the artery wall.

To overcome the challenge of the *in vivo* assessment of the mechanical properties of the arterial wall, the aforementioned original procedure encompasses (i) the quantification of the aneurysm deformation from an anatomical image by measuring the variation of luminal volume and (ii) using this quantity to identify the wall behaviour law parameters with an inverse analysis based on the finite element method. The evaluation of the mechanical properties of the aneurysm wall allows determining the stress state, which is a valuable measure in the rupture assessment. The procedure reliability was asserted through several tests of the device on different study subjects.

## 2. Material and methods

### 2.1. Creation of a saccular aneurysm on the rabbit carotid artery

**Figure 1.**
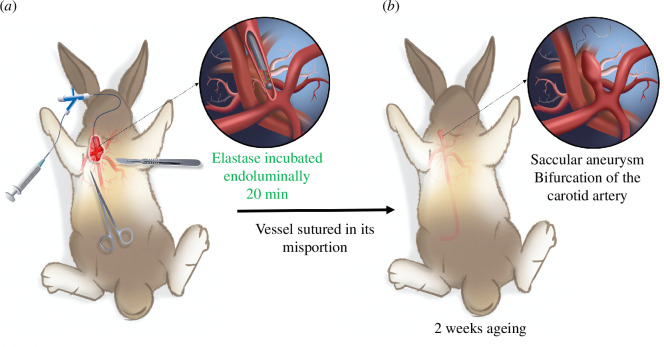
Creation of a saccular aneurysm on a rabbit carotid artery [32,33]. (*a*) Occlusion of the vessel origin and endoluminal incubation of elastase above the embolectomy catheter. Suture of the vessel in its midportion. (*b*) Resulting saccular aneurysm created two weeks ageing after the suture.

A saccular aneurysm on the bifurcation of the carotid artery of a rabbit was provoked to mimic a human intracranial aneurysm in terms of wall microstructure, the size of the vessels and the aneurysm. This kind of aneurysm typically forms in the Willis polygon area; the majority of aneurysm ruptures occur in this zone, where the mechanical stresses between the arterial wall and the blood flow are high [31]. An animal model of intracranial aneurysms suitable for testing endovascular devices was implemented following the work of Altes *et al*. and Cloft *et al*. [32,33]. The procedure was applied to six New Zealand female white rabbits (3–4 kg) and is shown in figure 1. For the creation of the aneurysm, anaesthesia was induced by intramuscular injection of ketamine and medetomidine (15/0.15 mg/kg) and maintained under gas anaesthesia (sevoflurane) using an endotracheal tube. The common right carotid artery was surgically exposed, and the vascular approach was performed using the Seldinger technique [34]. A 5 Fr Desilets–Hoffman catheter was passed 5 cm retrograde to the origin of the right common carotid artery. A 4 Fr Fogarty embolectomy catheter was advanced through the Desilets–Hoffman catheter until the origin of the right common artery and inflated with contrast agent (IOMERON 400, 400 mg iodine/ml). The occlusion of the vessel origin was controlled with fluoroscopic guidance and a contrast agent injected through the Desilets–Hoffman catheter. Elastase, a proteolytic enzyme catalysing the hydrolysis of elastin (Elastase, Porcine Pancreas, High Purity, Crystallized, CAS 39445-21-1), was incubated endoluminally in the proximal common carotid artery above the embolectomy catheter. It enabled the destruction of the elastic laminae, thereby preventing the arterial contraction that occurs after the occlusion. The elastase was infused in the arterial stump for 20 min for all the six rabbits. Then, the balloon of the embolectomy catheter was deflated, and the catheters were removed. The occlusion was removed under fluoroscopic guidance to check the condition of the artery. The vessel was sutured in its midportion, and the skin was sutured on the neck of the rabbit. Two weeks ageing time was observed before the device was tested on the created aneurysm. The animal experimentation was approved by the animal review committee of the institution (Cellule Animaux utilisés à des Fins Scientifiques (AFiS)) and authorized by the French Ministry of Higher Education, Research and Innovation (licence number APAFIS#36854).

### 2.2. *In vivo* application of the DDP and quantification of the aneurysm deformation

#### 2.2.1. Medical imaging and functional principle of the aneurysm DDP

For the application of the DDP [27] on the created aneurysms, anaesthesia was induced by intramuscular injection of ketamine and medetomidine (15/0.15 mg/kg) and maintained under gas anaesthesia (sevoflurane) using an endotracheal tube. The DDP is a 5 Fr angiographic catheter modified and diverted from its main use. It is connected to a computed tomography (CT) contrast media injector (MEDRAD^®^ Stellant) and instrumented with a pressure probe, giving precise data regarding the stimulus induced. It pumps a miscible fluid against the aneurysm wall that leads to its deformation. The DDP was introduced through the right femoral artery using the Seldinger technique [34] and coupled with the use of a guide catheter. The positioning of the DDP in the aneurysm cavity was controlled with fluoroscopic guidance. The principle was to carry out *in vivo* mechanical testing of the wall without a potential uncertainty linked to the mechanical load applied to the inner wall. The *in vivo* detection of pulsation in the UIA was performed by Hayakawa *et al*. [35] with 3D-CT angiography. Nevertheless, the quantification of the deformation and the link with a mechanical load was not possible based on the analysis of these clinical images. Using the DDP provides direct feedback on the load applied with the flow rate defined and the pressure measured, coupled with an estimation of the blood flow waveform. The temporal uncertainty that may occur between the clinical image acquisition and the cardiac cycle moments estimated [36] (with the rabbit heart rate, it was impossible to calibrate the image acquisition on the systole and the diastole for instance) is overcome by controlling and maintaining the DDP effect over time.

**Figure 2.**
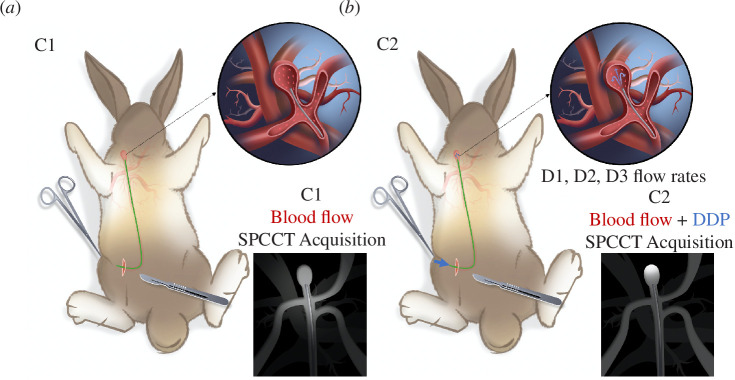
Experimental protocol of the DDP application. (*a*) C1: DDP positioned in the aneurysm without applying a flow rate and helical acquisition with the SPCCT (Philips Healthcare). (*b*) C2: application of a flow rate by the DDP on the aneurysm wall in addition to the blood flow. Flow rates of 150, 170 and 190 ml/min (D1, D2 and D3, respectively). Axial acquisition with the DDP for each flow rates. The contrast agent is injected exclusively through the DDP flow.

To test the DDP, the circulation and the aneurysm behaviour were monitored by the SPCCT prototype system (Philips Healthcare). This technology is emerging as a new and promising imaging modality in the field of CT X-ray imaging, owing to its energy-resolving detectors, known as photon counting detectors [37]. This modified base clinical system provides a spatial resolution down to 250 μm [29,30]. It was essential to reach this resolution to visualize the wall displacements which would not be possible with a standard CT scan. Indeed, the resulting deformation was expected to be in this order of magnitude based on the *in vitro* experimental studies and the numerical studies prior to the *in vivo* application [27]. It was mandatory to reach this scale of displacement to prevent the wall from breaking. The principle and the timeline of the image acquisition are shown in figure 2. Two configurations were considered for the image acquisition: Configuration 1 (C1) with the DDP positioned in the aneurysm without applying a flow rate, and Configuration 2 (C2) with applying a flow rate by the DDP on the aneurysm wall in addition to the blood flow. For C1, the acquisition sequence of the SPCCT was helical (50 mAs, rotation time 0.33 s, tube current 210 mA, tube voltage 120 kV, pitch 1.3) with a contrast agent injected through the ear vein (IOMERON 400, 400 mg iodine/ml). For C2, no contrast agent was injected through the ear vein; it was done exclusively through the DDP flow (IOMERON 400, 400 mg iodine/ml diluted at 10% in physiological liquid). Flow rates of 150, 170 and 190 ml/min (D1, D2 and D3, respectively) were applied for 3 s; for each stimulus, an axial acquisition (70 mAs, rotation time 0.33 s, tube current 210 mA, tube voltage 120 kV, pitch 1) was done 2 s after the triggering of the pulse to ensure the visualization of the effect of the device on the aneurysm. The uniqueness of the contrast agent in the device for C2 allowed for focusing exclusively on the aneurysm behaviour without artefacts potentially linked to the visualization of the blood flow in the surrounding arteries. The reconstruction of the conventional images was done with the Sharp filter: a 250 mm Field-of-View (FOV), a 1024 matrix and 0.25 mm voxels (slice thickness of 0.25 mm). The rabbits were sacrificed after the medical image acquisition.

#### 2.2.2. Quantification of aneurysm deformation: determining the luminal volume variation

**Figure 3.**
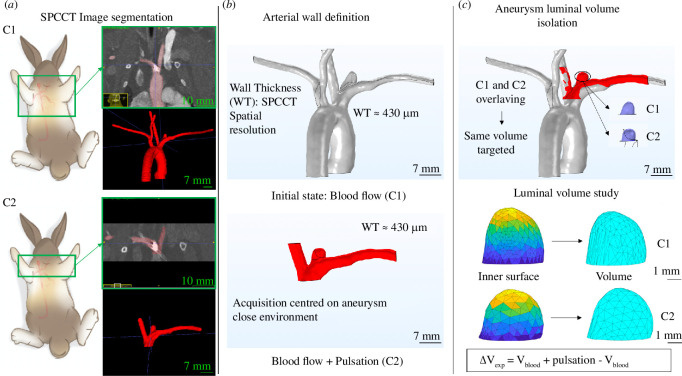
(*a*) SPCCT clinical imagery of C1 and C2. Frontal view of the acquisition area and segmentation of the artery/aneurysm flow with ITK Snap. (*b*) Artery wall building, combining three-dimensional Slicer and COMSOL Multiphysics geometry tools from the C1 and C2 segmentations. Homogeneous thickness on the artery and the aneurysm. (*c*) Luminal volume extraction from the C1 and C2 models combining COMSOL Multiphysics and Matlab. Overlaying of the C1 and C2 arterial walls to ensure the same volume extraction.

The inverse analysis procedure leading to the determination of the mechanical properties of the aneurysm wall [36] is based on the study of the luminal volume variation (mm^3^) of the aneurysm. An original procedure was implemented to extract this quantity from the SPCCT images. The studies of C1 and C2 images were carried out with the open-source software ITK Snap [38]; the segmentation of the aneurysm/artery flow was done with the semi-automatic method. Based on the segmented images, an original procedure combining three-dimensional Slicer, COMSOL Multiphysics geometry tools and Matlab was implemented to build an arterial wall and extract the C1 and C2 luminal volumes from the aneurysm inner surface (figure 3). This method was conceived to also be applicable to the luminal volume study in the inverse analysis procedure.

**Figure 4.**
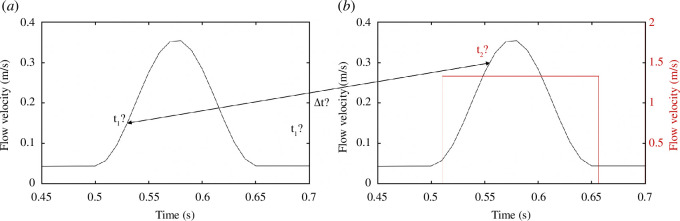
*a*: Flow velocity (m/s) in the rabbit carotid artery for a study subject derived from the measurement of the carotid blood flow rates for New Zealand white rabbits [39,40]. *b*: Application of a 170 ml/min stimulus with the DDP in addition to *a* to overlay the cardiac cycle. Unknown of the acquisition moment between *a* and *b* with the SPCCT.

As shown in figure 3, overlaying the C1 and C2 models was essential to ensure the extraction of the same aneurysm area based on the surrounding common elements. As the rabbit heart rate is too fast [39,40] to calibrate the SPCCT acquisition on this value (rotation time of 0.33 s), the precise cardiac cycle moments considered in C1 and C2 remained unknown. Figure 4 depicts this $\Delta t=|t_{1}-t_{2}|$ unknown between C1 and C2 for a rabbit cardiac cycle. This time-dependent flow velocity was designed from measurements of the carotid flow rates of New Zealand white rabbits [39,40] and applied to the specific study subject, the DDP stimulus overlaying this cycle. This limitation regarding time identification was taken into account in the inverse analysis procedure.

### 2.3. Identification of the aneurysm wall behaviour law parameters with inverse analysis

#### 2.3.1. Modelling the *in vivo* testing of the device with the finite element method

The inverse analysis was based on the numerical modelling of the experimental testing of the device. The model was implemented with the finite element method in COMSOL Multiphysics. It included the arterial wall and the DDP as solid mechanic elements and the blood flow and the DDP flow as fluid mechanics elements. The FSI between the flows and the arterial wall was studied.

##### 2.3.1.1. Mechanical laws and boundary conditions applied to the artery wall and the DDP

The DDP domain was built as a hollow cylinder with an inner diameter of 1.68 mm and an outer diameter of 1.87 mm. The DDP material was PTFE modelled with an isotropic linear elastic model, a Young’s modulus of E=0.41 GPa and a Poisson ratio of ν=0.46 . The DDP extremities were embedded; the movement of the device induced by the blood flow was not taken into account. The arterial wall definition was built from the SPCCT image segmentation with the original procedure depicted in figure 3. A uniform thickness of 430 μm was defined, which was in line with the values reported in the literature (wall thicknesses between 170 and 680 μm was reported [41]). Drawing from a large number of sources, a hyperelastic material law was defined from studies of artery mechanical responses [42–45]. In this study, an isotropic incompressible Fung-like material law was adopted with ρ=1050 kg m^−3^ and the strain-energy function:


(2.1) $$W=\frac{a}{b}\left(exp\left(\frac{b}{2}(I_{1}-3)\right)-1\right),$$

where *I*_1_ is the first invariant of the right Cauchy–Green deformation tensor and *a* and *b* are material constants. This material law was already applied in the FSI numerical model of the aneurysm wall mechanical behaviour [46,47] and was also used in an inverse analysis procedure based on the luminal volume variation of aortic aneurysms [36]. In the literature, it was reported that for UIA, *a* = 353.6 kPa and *b* = 16.7 [46,47]; whereas *a* = 176.8 kPa and *b* = 16.7 were identified experimentally as parameters associated with ruptured aneurysms [18,41,47]. These values were used to define a range for the inverse analysis procedure. Regarding the boundary conditions, a zero displacement condition was applied to the extremities of the artery body [48,49]. It was reported that this condition did not affect the stress and stretch of the aneurysm sac [47].

##### 2.3.1.2. Boundary conditions and modelling of the DDP and the blood flows

A single fluid mechanics component included the artery blood flow (Fluid 1) and the flow delivered by the DDP (Fluid 2). It was assumed that Fluids 1 and 2 were miscible and could be defined as a unique single-phase flow with the solid DDP boundaries separating and defining two distinct inputs. Blood was assumed to be Newtonian since the non-Newtonian assumption was without ambivalence for vessel diameters smaller than 0.1 mm [50], which was not reached for the arteries studied (considering all the study subjects, the minimum diameter for artery branches was 0.9 mm) . The flow was defined as an incompressible, laminar and thus Newtonian flow with a constant density of 1050 kg m^−3^ and a dynamic viscosity of 0.0035 Pa s [46–48,51,52] following the Navier–Stokes equations:


(2.2) $$\rho \frac{\partial {\text{u}}_{fluid}}{\partial t}+\nabla \cdot \eta (\nabla {\text{u}}_{fluid}+(\nabla {\text{u}}_{fluid}{)}^{T})+\rho ({\text{u}}_{fluid}.\nabla ){\text{u}}_{fluid}+\nabla P=\text{F},$$

(2.3) $$\nabla \cdot {\text{u}}_{fluid}=0.$$

With the velocity field denoted ${\text{u}}_{fluid}$ , the density, ρ , the dynamic viscosity, η , the pressure P and a body force term such as gravity, **F**. A simplified pulsatile waveform was applied to the Fluid 1 inlet, as shown in figure 4. This profile did not match perfectly with the recorded *in vivo* flow profiles [39,40] but was used classically in the literature [51,53]. Nevertheless, the velocity values associated with each study subject at the systole peak were consistent with the literature (0.35 m/s for the present study, as velocities of 0.4 m/s were reported [39,40]). A coupled pressure pulsatile profile was imposed at the artery outlets with a range from 43 to 104 mmHg (5732 to 13 865 Pa) [54]. A pulsatile profile was applied to the Fluid 2 inlet (as shown in figure 4). The Fluid 2 flow rates were considered to be in line with the experimental DDP applications: 150, 170 and 190 ml/min (D1, D2 and D3, respectively).

##### 2.3.1.3. Numerical modelling of the interaction between the DDP flow and the aneurysm wall

The FSI multiphysics coupling combined the Fluid 1 + 2 flow with the artery/aneurysm wall mechanics. The FSI couplings appeared on the inner wall of the artery which was the boundary between the flow (Fluid 1 + 2) and the artery. The physics interface used an arbitrary Lagrangian Eulerian (ALE) method [46,48,51,52,55] to combine the Fluid 1 + 2 flow formulated using an Eulerian description, and a spatial frame with artery solid mechanics formulated using a Lagrangian description and a material reference frame. The one-way fluid−solid interaction [47,51] solved for the fluid flow, computed the load and then applied it to the solution for the artery wall displacements sequentially. The coupling was therefore unidirectional, and this strategy was chosen to avoid the numerical instabilities that arise in the numerical solution for flows in arterial geometries. This allowed the implementation of a large number of numerical models for the inverse analysis: for each rabbit tested, 51 models in C1 were necessary and 51 models in C2 at D1, D2 and D3. Furthermore, this work focused primarily on the mechanical response of the wall; hence, simulating it only with the blood flow and DDP forces was sufficient for the comparative analysis.

### 2.3.2. Inverse analysis procedure used to identify the mechanical properties of the aneurysm wall

**Figure 5.**
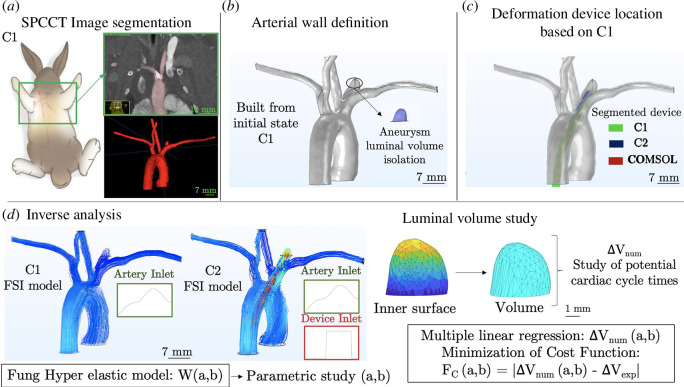
Original inverse analysis procedure was developed for the identification of the mechanical properties of the arterial wall. (*a*) Segmentation of the C1 SPCCT images. (*b*) Building of a 430 μm thickness based on the C1 segmentation. Delimitation of the same aneurysm volume compared to the experimental analysis. (*c*) Implementation of the DDP numerical model based on the C2 segmentation. (*d*) Parametric study performed on the fluid–structure identification model leading to the definition of a $\Delta V_{num}(a,b)$ function and a cost function $F_{C}(a,b)$ to minimize.

A computational model of the artery and the DDP was built from the segmentation of the C1 SPCCT images. The arterial wall was built in accordance with the procedure depicted in figure 3 with a uniform thickness of 430 *μ*m. The DDP model was also built according to its associated segmentation (figure 5). The same aneurysm volume compared to the experimental variation study was isolated, and the same procedure was applied to extract its value. The FSI between the flows and the aneurysm wall was computed in C1 and C2 with the two-element (*a*,*b*) hyperelastic model. A parametric study was performed for each study subject with 51 (*a*,*b*) couples considered for C1, C2 and in each case D1, D2 and D3: based on the literature data, a ranged between 85 and 645 kPa, b ranged between 14.4 and 17.6 [18,41,46,47]. Assuming that the luminal volume variation $\Delta V_{num}$ depends only on the model parameters, a volumetric function can be defined as:


(2.4) $$\Delta V_{num}(a,b)=\alpha_{1}a^{2}+\alpha_{2}b^{2}+\alpha_{3}a+\alpha_{4}b+\alpha_{5}ab+\alpha_{6},$$

with the $\alpha_{i}$ coefficients derived from a multiple linear regression based on the least-square method [36]. The computation and the regression were performed for the D1, D2, D3 flow rates on each study subject. A cost function was defined between $\Delta V_{num}$ and $\Delta V_{exp}$ :


(2.5) $$F_{C}(a,b)=|\Delta V_{num}(a,b)-\Delta V_{exp}|.$$

For each flow rate in C2, the couple of parameters minimizing $F_{C}$ were identified. What is more, as shown in figure 4, there was a temporal unknown with $\Delta V_{exp}$ : several potential Δ *t* were considered for each study subject at every flow rate ( 0<Δt<0.07 s with a 0.01 s increment). For each Δ *t,* the associated $\Delta V_{num}$ and $F_{C}$ were studied to find the one with the lowest minimum.

To quantify the aneurysm stress state for the model parameters identified, the first principal Cauchy stress field of the aneurysm inner wall $\sigma_{1}$ was considered [47]. The $<\sigma_{1}>$ mean of the aneurysm area was computed for the C2 and compared to the C1. The mean pressure <P> applied on the aneurysm wall was also considered in C1 and C2.

## 3. Results

### 3.1. Creation of the aneurysm and experimental luminal volume variation computation

Among the six rabbits tested in this study, three aneurysms were successfully created, among which two were associated with exploitable medical images (denoted as Sample 1 (S1) and Sample 2 (S2)). The luminal volumes (mm^3^) extracted from the segmentation of the SPCCT images and the associated experimental volume variation $\Delta V_{exp}$ between C1 and C2 with flow rates applied by the DDP are detailed in table 1. When considering the results of S1 and S2, the maximum $\Delta V_{exp}$ was 1.03 mm^3^ reached with S2 and D3 (equivalent to the variation $\Delta V_{exp-S2-D3}=\frac{V_{C2-D3}-V_{C1}}{V_{C1}}=10.5\;\%$ , with the experimental luminal volumes $V_{C1}$ reached in C1 and $V_{C2-D3}$ reached at D3); the minimum was 0.12 mm^3^ reached with S1 and D1 (equivalent to $\Delta V_{exp-S1-D1}=\frac{V_{C2-D1}-V_{C1}}{V_{C1}}=0.92\;\%$ , with the experimental luminal volumes $V_{C1}$ reached in C1 and $V_{C2-D1}$ reached at D1).

**Table 1.** Experimental luminal volumes (mm^3^) extracted from the segmentation of the SPCCT images and associated variation $\Delta V_{exp}$ between C1 and C2 with the flow rates applied with the DDP: D1 = 150 ml/min, D2 = 170 ml/min and D3 = 190 ml/min (C2–D1, C2–D2 and C2–D3, respectively).

	C1	C2–D1	C2–D2	C2–D3
S1				
luminal volume (mm^3^)	12.98	13.10	13.86	13.57
$\Delta V_{exp}\;(mm^{3})$		0.12	0.88	0.59
S2				
luminal volume (mm^3^)	9.81	10.64	10.38	10.84
$\Delta V_{exp}\;(mm^{3})$		0.83	0.57	1.03

### 3.2. Identification of the mechanical parameters of the aneurysm wall

**Figure 6. F6:**
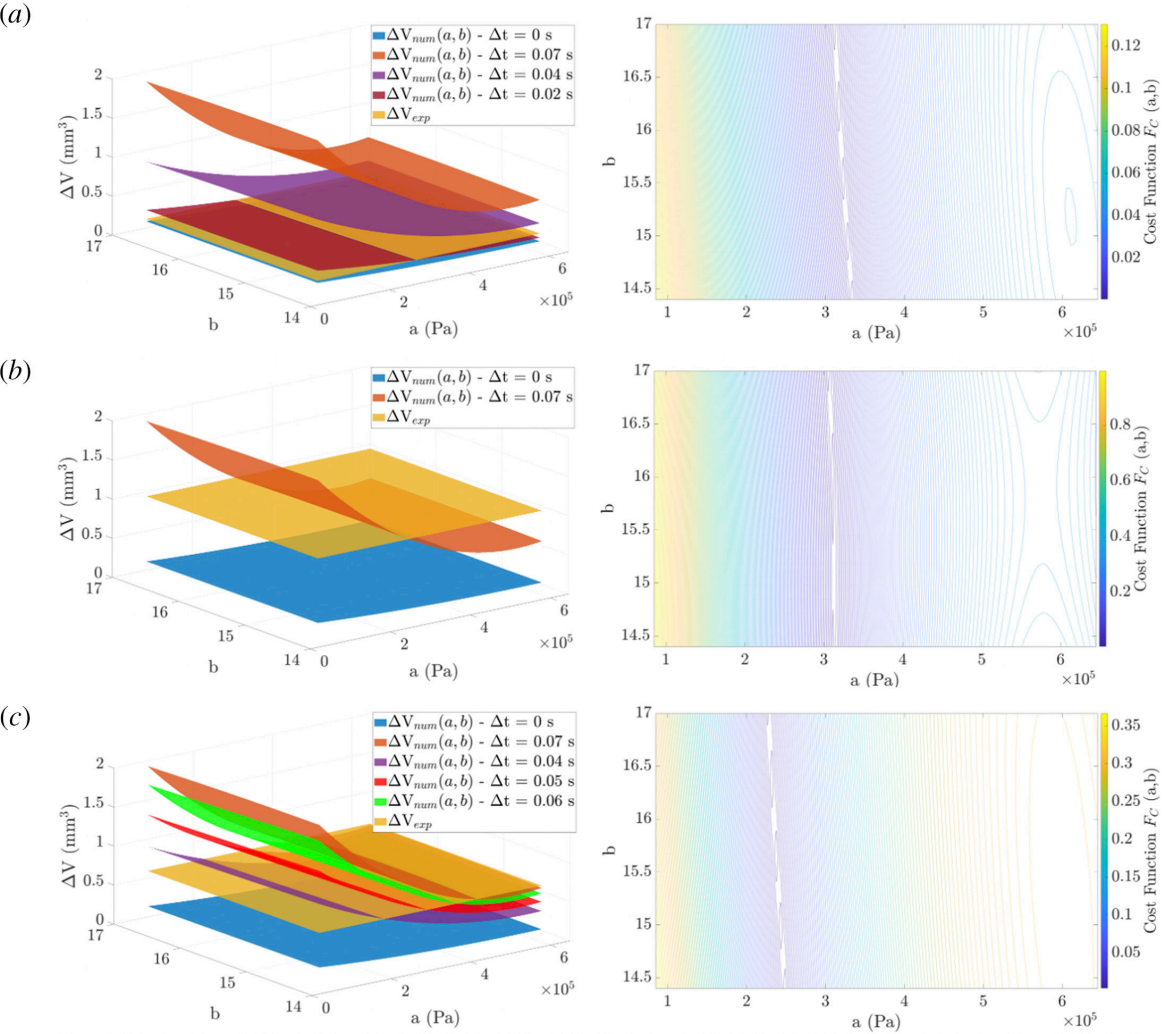
S1 analysis: (Left) Multiple linear regressions were associated with all the most relevant $\Delta t=|t_{1}-t_{2}|$ considered. Experimental luminal volume variation $\Delta \,V_{e\,x\,p}$ (yellow) intersects with the numerical variation $\Delta \,V_{n\,u\,m}$ and specific Δ *t*. (Right) The Cost Function for the Δ *t* enables reaching the minimum. (*a*) D1 = 150 ml/min, Δ *t* = 0.02 s. (*b*) D2 = 170 ml/min, Δ *t* = 0.07 s. (*c*) D3 = 190 ml/min, Δ *t* = 0.04 s.

**Figure 7. F7:**
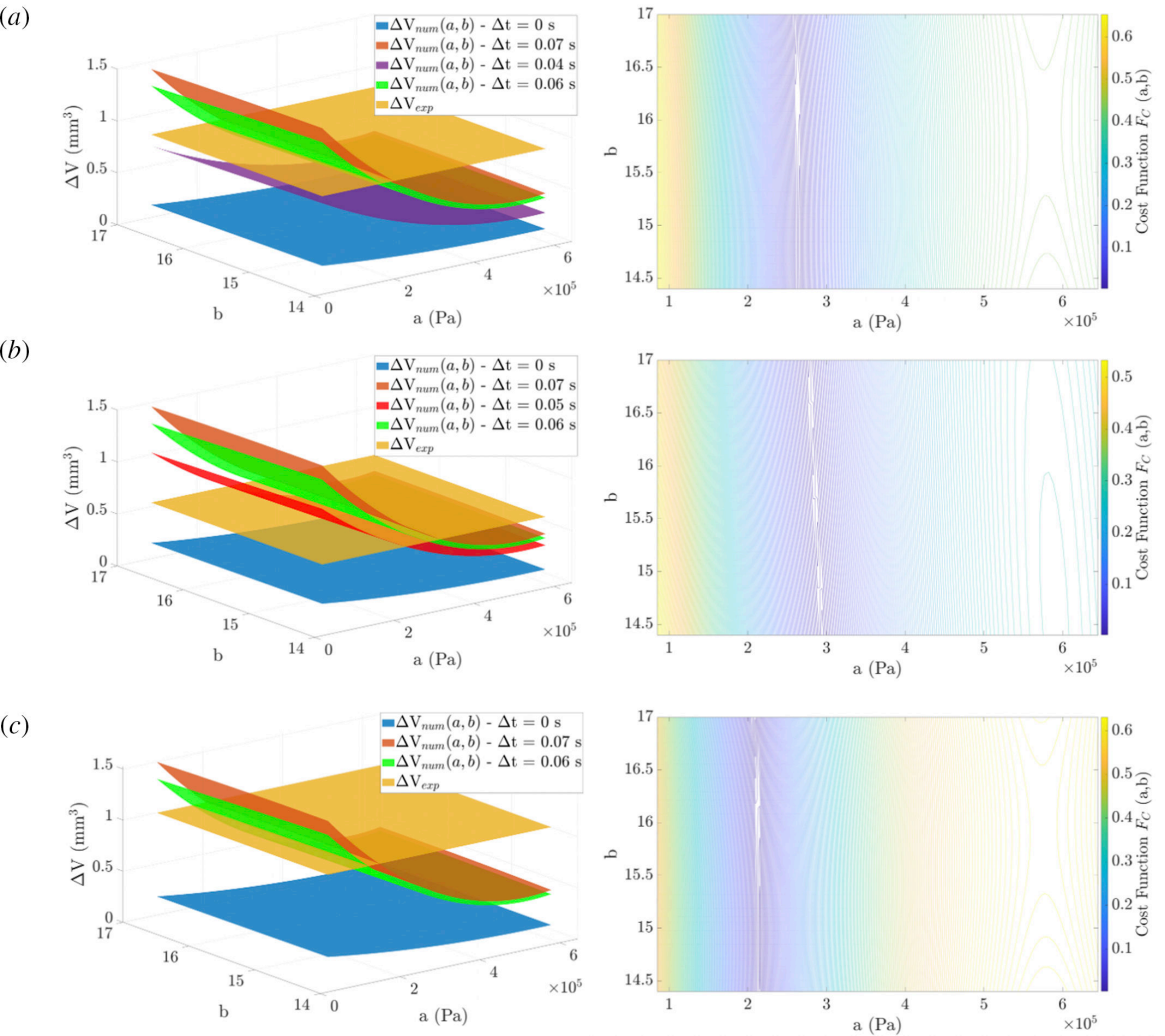
S2 analysis: (Left) Multiple linear regressions were associated with all the most relevant Δ *t* considered. Experimental luminal volume variation $\Delta \,V_{e\,x\,p}$ (yellow) intersects with the numerical variation $\Delta \,V_{n\,u\,m}$ and specific Δ *t*. (Right) The Cost Function for the Δ *t* enables reaching the minimum. (*a*) D1 = 150 ml/min, Δ *t* = 0.07 s. (*b*) D2 = 170 ml/min, Δ *t* = 0.05 s. (*c*) D3 = 190 ml/min, Δ *t* = 0.07 s.

The multiple linear regression results are shown in figures 6 and 7 [Fig F6 F7]for S1 and S2, respectively. Regarding the S1 analysis, at D1 = 150 ml/min the minimum was reached for Δt = 0.02 s, at D2 = 170 ml/min the minimum was reached for Δt = 0.07 s and at D3 = 190 ml/min the minimum was reached for Δt = 0.04 s. Regarding the S2 analysis, at D1 = 150 ml/min the minimum was reached for Δt = 0.07 s, at D2 = 170 ml/min the minimum was reached for Δt = 0.05 s and at D3 = 190 ml/min the minimum was reached for Δt = 0.07 s. The Cost Functions associated with each identified Δt are directed towards the material constants detailed in table 2.

**Table 2.** Material constants of the hyperelastic material law identified for S1 and S2 with the original inverse analysis procedure based on the luminal volume variation and the DDP flow rates applied experimentally.

	D1 = 150 ml/min	D2 = 170 ml/min	D3 = 190 ml/min
S1			
*a* (kPa)	316.92	311.26	232.07
*b*	16.21	16.13	16.37
S2			
*a* (kPa)	260.35	283.98	209.44
*b*	16.87	16.13	16.82

For the material constants identified, the variation of luminal volume $\Delta V_{num/mat}$ between C1 and C2 was associated with each flow rate (D1, D2, D3) and Δt was computed numerically with the associated model. The relative error e of $\Delta \,V_{n\,u\,m/m\,a\,t}$ compared to $\Delta \,V_{e\,x\,p}$ was computed for each model $\left(e=\frac{|\Delta V_{num/mat}-\Delta V_{exp}|}{\Delta V_{exp}}\right)$ . For S1 and D1, D2, D3, e was 4.33%, 3.41% and 3.86%, respectively. For S2 and D1, D2, D3, e was 8.84%, 8.98% and 7.78%, respectively.

### 3.3. Quantification of the aneurysm stress state

**Figure 8.**
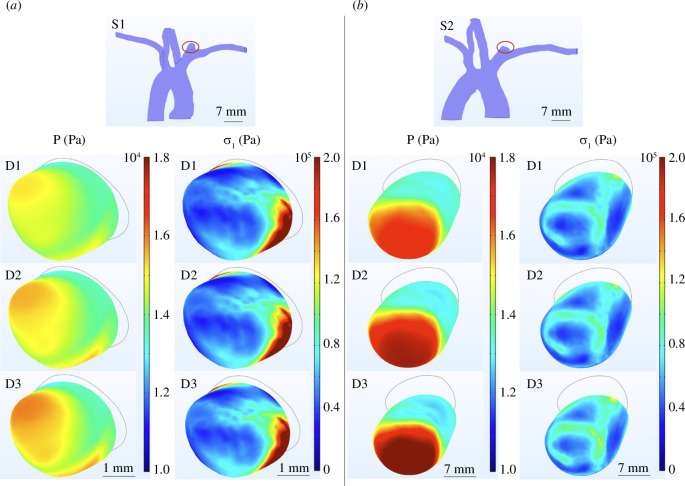
Stress state and pressure analysis of S1 (left) and S2 (right): retrieval of the aneurysm area, pressure P (Pa) applied on the aneurysm inner wall in C2 at the systole peak for D1 = 150 ml/min, D2 = 170 ml/min, D3 = 190 ml/min, first principal Cauchy stress $\sigma_{1}$ (Pa) resulting on the inner wall in C2 at the systole peak for D1, D2, D3.

Using the identified parameters of the material law, a numerical study of the aneurysm wall based on the first principal Cauchy stress and the pressure applied was performed. The mean first principal Cauchy stress on the aneurysm area $<\sigma_{1}>$ , the mean pressure applied on the aneurysm area inner wall <P>, the stress variation $\Delta <\sigma_{1}>=\frac{<\sigma_{1}^{C2}>-<\sigma_{1}^{C1}>}{<\sigma_{1}^{C1}>}$ (with $<\sigma_{1}^{C1}>$ and $<\sigma_{1}^{C2}>$ the mean stress at the systole peak (kPa) in C1 and C2, respectively) and the pressure variation $\Delta <P>=\frac{<P_{C2}>-<P_{C1}>}{<P_{C1}>}$ (with $<P_{C1}>$ and $<P_{C2}>$ the mean pressure at the systole peak (kPa) in C1 and C2, respectively) are detailed in table 3 for S1, S2 during C2 with D1, D2, D3 at the systole peak. The stress field and the pressure applied on the aneurysm inner wall are shown in figure 8 for D1, D2 and D3 in C2 at the systole peak.

**Table 3.** Numerical analysis of S1 and S2: mean pressure (kPa) and mean first principal Cauchy stress (kPa) applied on the aneurysm area at the systole peak in C2 with the addition of D1 = 150 ml/min, D2 = 170 ml/min, D3 = 190 ml/min (C2–D1, C2–D2, C2–D3, respectively). Mean pressure and first principal stress variation Δ<P> and $\Delta <\sigma_{1}>$ between C1 and C2 at the systole peak in C2 with D1, D2 and D3 (C2–D1, C2–D2 and C2–D3, respectively).

	C2–D1	C2–D2	C2–D3
S1			
<P> (kPa)	14.2	14.3	14.5
Δ <P> (%)	3.17	4.12	5
<σ_1_> (kPa)	88.2	88.9	92.5
∆ < σ_1_> (%)	2.55	3.35	4.45
S2			
<P> (kPa)	14.8	15.1	15.4
∆ <P> (%)	7.09	9.38	11.35
< σ_1_ > (kPa)	64	64.4	69.1
∆ < σ_1_ > (%)	6.08	8.07	10.59

## 4. Discussion

The key aim of this study was to determine the mechanical properties of an intracranial aneurysm model in a small animal through the *in vivo* application of an original device. Thus, for a known mechanical load on the aneurysm wall, it would be possible to estimate the stress state and assess the rupture risk. In response to the effect of the DDP, it was possible to quantify a variation of the aneurysm luminal volume between C1 and C2 at the D1, D2, D3 flow rates (table 1). Nevertheless, as shown in figure 4, the Δt between C1 and C2 could be different in each case, so much so that it was impossible to validate a trend in the values (such as an increase of ΔV related to flow rates). For S2 at the flow rates D1, D3, the Δt was similar and an increase of ΔV was noticed (from 0.83 to 1.03 mm^3^), confirming expectations. The use of the SPCCT as medical imaging for the quantification of the luminal volume variation was extremely relevant and original thanks to the high spatial resolution [29,30,37]. Although the Δt remained an immutable unknown for the rabbit animal model, it would be possible to overcome this limitation with different animals and a slower heart rate. This promising technique may enable further modifications to the procedure to improve the quantification of the DDP effect (in terms of contrast agent injection and acquisition sequence to facilitate the visualization of the aneurysm in C1, for instance).

With respect to the bibliography and for an isotropic incompressible Fung-like material law applied to the artery wall with material constants a and b : for UIA, a = 353.6 kPa and b = 16.7 were reported [46,47]; a = 176.8 kPa and b = 16.7 were identified experimentally as parameters associated with ruptured aneurysms [18,41,47]. The material constants detailed in table 2 were congruent with these data for S1 and S2, which validated the proposed *in vivo* parameter identification procedure. For unruptured aneurysms, the material constants remained in the same order of magnitude and higher than the parameters identified for ruptured aneurysms. Furthermore, for S1 and S2, consistency was observed with the material constants found from D1, D2 and D3 and the associated volume variation for the same study subject. Moreover, when the weakening of the aneurysm wall was associated with a decrease in the material constant a according to the literature [18,41,47], this phenomenon could be observed chronologically. When increasing DDP flow rates were applied consecutively in time, the material constants a were lower for D3 compared to D1 and D2 (noticed for S1 and S2, with decreases during the application of D2 to D3 of 25% and 26%, respectively): a weakening of the aneurysm wall owing to successive and increasing mechanical load could be intuited. What is more, the relative errors between the $\Delta \,V_{n\,u\,m/m\,a\,t}$ (numerically computed for the identified material constants) and the measured $\Delta V_{exp}$ were low (between 3.41% and 8.98%) and congruent with the computed errors of inverse analysis methods (errors ranging between 6.5% and 19.32% were reported for a biomechanical liver model [56], and 0.6% was reported for an aortic aneurysm [36]) which also validated the constants identified. Moreover, Trabelsi *et al.* [36] reported aortic aneurysm luminal volume variations based on CT scan observation between 1.80% and 18.76% for Δt=0.4 s (systole versus supposed diastole) and between 0.99% and 11.27% for Δt=0.2 s (systole versus supposed mid cycle). The computed luminal volume variations were between 0.92% and 10.5% and were consistent with this study even though it was a different kind of aneurysm.

Wall samples of the aneurysms were collected after the procedure: further mechanical tests such as indentation or tensile tests may provide additional validations of these constants. Those samples may also be used to precisely determine the IA wall thickness. Thanks to the material constants identified, the associated stress state will be used to determine the rupture criterion of the aneurysm. With the numerical study performed using the constants identified, it was possible to quantify the stress applied on the aneurysm wall in addition to the blood flow (with stress variation between 2.55% and 10.59%) for a known solicitation. These data are valuable since it will always be more achievable to have a precise value of the flow rate applied with the DDP rather than a measurement of the blood flow made simultaneously with the volume study (especially in the case of intracranial blood flow). The mean values of these quantities on the aneurysm area also linked the solicitation to the shape of the aneurysm. A ratio linking the pressure applied to the aneurysm wall (linked to the DDP flow rates) and the proper combination of all the stress components will be extracted for each DDP test (including, the values detailed in table 3). It will be compared to that associated with the experimental evidence of the aneurysm rupture, also caused by a known solicitation. However, the experimental procedure must be modified and improved to efficiently characterize the rupture without causing unnecessary animal suffering.

Nevertheless, the procedure and especially the numerical model presented in this study presented certain limits. The inlet and outlet conditions applied in the fluid mechanics computation were not based on measurements made on S1 and S2 in particular. The flow rates and pressure profiles were based on the literature [39,40,54] and adapted to the specific branch sections of S1 and S2. The velocity values at the systole peak were consistent with the literature for carotid artery measurements on similar rabbit species and build. However, the flow measurements under real-time control [39,40] in addition to the existing experimental procedure (containing the medical image acquisition and the DDP testing) may add too many complexities. The inverse analysis procedure based on these first results could also be refined, especially with the mechanical characterization focused on the aneurysm area. Indeed, a simplification of the procedure described was a homogeneous distribution of the material constants on the artery and the aneurysm area. On the strength of these identified constants, a second phase could be applied to the aneurysm area to highlight a potential heterogeneity in terms of mechanical properties between the artery and the aneurysm. Furthermore, refining the numerical model and the theoretical assumptions may be a means of improving the procedure. A single-layer artery with an isotropic material was considered for this model, but multiple-layer anisotropic hyperelastic materials have also been proposed [42,57,58]. Local heterogeneities of the thickness and mechanical properties of the aneurysm walls could also be added to get close to clinical observations [47]. Indeed, since IA rupture areas of 1 mm^2^ were reported [59], it could be interesting to refine the mechanical properties following such dimensions in the IA volume. Models of blood flow turbulence into intracranial aneurysms could also be a significant addition, although not widely used in the literature [60]. Finally, the accurate numerical reproduction of the experimental DDP remains complicated, even based on analysed during the current study are its visualization via clinical imaging. The potential movement of the DDP induced by the Fluid 1 and 2 flows was not taken into account in the numerical modelling, even if it were very unlikely in view of the aneurysm size.

## 5. Conclusion

The *in vivo* assessment of the wall mechanical properties using an animal model of intracranial aneurysms was performed by implementing an original procedure based on the visualization of the aneurysm deformation induced by a DDP. The imaging of the deformation was ensured by the SPCCT (Philips Healthcare), which provides an extremely high spatial resolution. The material constants were identified by an inverse analysis procedure based on an FSI finite element method and using the quantification of the aneurysm luminal volume variation. This procedure was successfully performed and validated on two animal samples, with, in each case, the aneurysm being subjected to several mechanical loads induced by the DDP. The evaluation of the mechanical properties of the aneurysm wall was allowed for the determination of the stress state, which is a valuable measure in the rupture assessment. Based on these results, a criterion linking the stress state and the pressure applied to the aneurysm wall was defined.

The next steps encompass the enhancement of the experimental procedure to provide valuable data regarding the rupture. The numerical model used in the inverse analysis could also be refined to get closer to clinical reality. The long-term objective remains to provide clinicians with a non-invasive patient-specific decision tool to predict the rupture of an IA from a standard clinical image. The probability of rupture will be determined by a machine learning algorithm linking the aneurysm shape observed clinically with a database containing information on the mechanical properties and rupture characterizations of aneurysms. The results of the present study provide invaluable information for achieving this objective.

## Data Availability

The datasets generated during and/or analysed during the current study are available from the corresponding author on reasonable request [61].
